# Unique Directional Motility of Influenza C Virus Controlled by Its Filamentous Morphology and Short-Range Motions

**DOI:** 10.1128/JVI.01522-17

**Published:** 2018-01-02

**Authors:** Tatsuya Sakai, Hiroaki Takagi, Yasushi Muraki, Mineki Saito

**Affiliations:** a Department of Microbiology, Kawasaki Medical School, Kurashiki, Okayama, Japan; b Department of Physics, School of Medicine, Nara Medical University, Kashihara, Nara, Japan; c Division of Infectious Diseases and Immunology, Department of Microbiology, School of Medicine, Iwate Medical University, Yahaba, Iwate, Japan; St. Jude Children's Research Hospital

**Keywords:** hemagglutinin-esterase-fusion glycoprotein, human influenza, influenza C virus, virus motility

## Abstract

Influenza virus motility is based on cooperation between two viral spike proteins, hemagglutinin (HA) and neuraminidase (NA), and is a major determinant of virus infectivity. To translocate a virus particle on the cell surface, HA molecules exchange viral receptors and NA molecules accelerate the receptor exchange of HA. This type of virus motility was recently identified in influenza A virus (IAV). To determine if other influenza virus types have a similar receptor exchange mechanism-driven motility, we investigated influenza C virus (ICV) motility on a receptor-fixed glass surface. This system excludes receptor mobility, which makes it more desirable than a cell surface for demonstrating virus motility by receptor exchange. Like IAV, ICV was observed to move across the receptor-fixed surface. However, in contrast to the random movement of IAV, a filamentous ICV strain, Ann Arbor/1/50 (AA), moved in a straight line, in a directed manner, and at a constant rate, whereas a spherical ICV strain, Taylor/1233/47 (Taylor), moved randomly, similar to IAV. The AA and Taylor viruses each moved with a combination of gradual (crawling) and rapid (gliding) motions, but the distances of crawling and gliding for the AA virus were shorter than those of the Taylor virus. Our findings indicate that like IAV, ICV also has a motility that is driven by the receptor exchange mechanism. However, compared with IAV movement, filamentous ICV movement is highly regulated in both direction and speed. Control of ICV movement is based on its specific motility employing short crawling and gliding motions as well as its own filamentous morphology.

**IMPORTANCE** Influenza virus enters into a host cell for infection via cellular endocytosis. Human influenza virus infects epithelial cells of the respiratory tract, the surfaces of which are hidden by abundant cilia that are inactive in endocytosis. An open question is the manner by which the virus migrates to endocytosis-active domains. In analyzing individual virus behaviors through single-virus tracking, we identified a novel function of the hemagglutinin and esterase of influenza C virus (ICV) as the motility machinery. Hemagglutinin iteratively exchanges a viral receptor, causing virus movement. Esterase degrades the receptors along the trajectory traveled by the virus and prevents the virus from moving backward, causing directional movement. We propose that ICV has a unique motile machinery directionally controlled via hemagglutinin sensing the receptor density manipulated by esterase.

## INTRODUCTION

**FIG 1:**
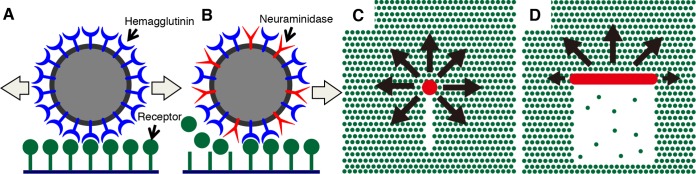
Hypotheses for influenza virus movement orientation mechanisms. (A) IAV moves on a cell surface by the HA-receptor exchange mechanism. (B) IAV NAs degrade receptor molecules along the pathway traveled by the virus, preventing the virus from moving backward and setting the direction of virus movement as forward. (C) A spherical IAV tends to move randomly. Receptor molecules are degraded by viral NAs in a narrow line along the virus trajectory. Because most receptors remain around the virus, the virus can move in all directions except for directly backward, resulting in a pattern of spherical virus movement that resembles random. (D) A filamentous ICV has the potential to move directionally. Receptors are two-dimensionally degraded by the ESs of ICV, which correspond to the NAs of IAV. The higher density of receptor molecules in front of the virus makes the virus move forward.

Virus motility is a major determinant for virus infectivity, and a novel motile mechanism was recently found in influenza A virus (IAV) (1). Utilizing this motile mechanism, IAV moves across the cell surface and migrates to target cells or specific membrane domains, where the virus enters into the target cell via cellular endocytosis. Compared with the motile mechanisms of bacterial or eukaryotic cells, this virus motile mechanism is very simple and is operated by just two viral spike proteins, hemagglutinin (HA) and neuraminidase (NA). HA binds to a viral receptor, either a sialoglycoprotein or sialoglycolipid, and NA, which is a hydrolytic enzyme, cleaves sialic acid from viral receptors (2, 3). Generally, HA functions in the virus attachment to the cell surface for virus infection, and NA functions in the release of progeny virus from host cells. In the IAV motile mechanism, HA translocates the virus particle on the cell surface via an HA-receptor exchange mechanism, in which the iterative association and dissociation between HA molecules and receptors work as the driving mechanism of virus movement (Fig. 1A) (1). NA makes it easier for the virus particle to move by suppressing the number of HA-receptor cross-bridges. If NA degrades the receptors on the pathway that the virus has already traveled, it can potentially prevent the virus from going backward (Fig. 1B). Consequently, influenza viruses do not return to regions where the viruses have previously existed but instead move into new regions. Interestingly, these mechanisms cause the virus to behave as if it has a memory of where it has been; through degrading and binding to the receptor molecules, NA and HA, respectively, contribute to this virus “memory” as writing and reading devices.

The directional movement of influenza virus appears to be biologically significant, allowing the virus to actively move away from regions where the virus did not undergo endocytosis so it can efficiently arrive at regions that are active in endocytosis. Although the attachment of influenza virus to cell surfaces recruits the endocytosis machinery, such as clathrin cargo, and induces endocytosis in cultured cells (4–6), endocytosis does not occur over entire cell surfaces *in vivo*, so virus motility is significant for virus infection. Notably, human influenza virus infects epithelial cells in the respiratory tract, the surface of which is covered by abundant cilia. Cilium membranes are inactive in endocytosis, and the endocytosis-active domains of ciliated cells are located in the cilium bases (7). Directional movement by the virus has likely advantages for its migration from the top to bottom along cilia. In addition to migration on a single cell surface, human influenza virus can require cell-to-cell migration because the human virus preferentially infects nonciliated cells, which are hidden by the cilia of neighboring ciliated cells (8). Like human influenza virus, avian influenza virus also requires cell-to-cell migration for virus infection. This requirement is due to avian influenza virus preferentially infecting epithelial cells at the bottom of the intestinal crypt, which is a deep invagination of the intestine luminal surface (9). Therefore, directional movement would likely provide advantages in allowing influenza virus to move away from the endocytosis-inactive surfaces and migrate to the target cells or cellular domains. However, the movement pattern of IAV is random rather than directional, and NA appears to be incapable of specifically orienting virus movement forward (1). In this study, we examined influenza C virus (ICV) motility to determine whether other types of influenza virus perform oriented movement in a way that is different from the random movement of IAV.

ICV usually has a filamentous morphology, and it has one spike protein called hemagglutinin-esterase-fusion glycoprotein (HEF) (2, 10, 11). The HA domain of HEF binds to a viral receptor, which is either a sialoglycoprotein or a sialoglycolipid with 9-O-acetyl-N-acetylneuraminic acid (Neu5,9Ac2)-terminated polysaccharides. The esterase (ES) domain of HEF is a 9-O-acetylesterase that cleaves O-linked acetyl groups at a C-9 position from Neu5,9Ac2 and functionally corresponds to the NA of IAV. We hypothesized that filamentous ICV would tend to move unidirectionally, unlike the spherical IAV (Fig. 1C and D). In IAV, the receptor molecules around a spherical IAV particle are degraded by viral NAs in a narrow line along the virus trajectory. Because most receptors remain around the virus, the virus can move in any direction except for directly backward, resulting in a pattern of spherical virus movement that resembles random. In contrast, the ESs of filamentous ICV degrade receptors two-dimensionally, and the relatively high density of receptor molecules in front makes the virus move forward in a straight path.

**FIG 2:**
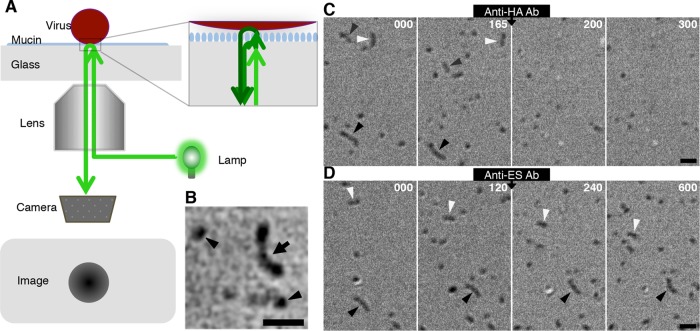
A novel imaging technique for analysis of virus motility. (A) Optics of SRICM. SRICM enhances the interference of light reflected by an interphase between the glass surface and virus particle and allows the imaging of unlabeled influenza viruses. (B) Representative SRICM images of unlabeled ICVs are shown. A filamentous ICV is observed as a rodlike object (arrow). Spherical ICVs are also seen (arrowheads). Bar, 1 μm. (C) Effects of anti-HA antibody on ICV movements. Three filamentous ICVs (black, gray, and white arrowheads) moved on the mucin-coated glass surface before the addition of anti-HA antibody. Representative images from various time points before (0 and 165 s) and after antibody addition (200 and 300 s) are shown. Time (in seconds) is indicated in the upper right of each panel. Bar, 1 μm. (D) Effects of anti-ES antibody on ICV movements. Two filamentous ICVs (black and white arrowheads) moved on the mucin-coated glass surface before the addition of anti-ES antibody. Representative images from various time points before (0 and 120 s) and after antibody addition (240 and 600 s) are shown. Time (in seconds) is indicated in the upper right of each panel. Bar, 1 μm.

To test our hypothesis, we investigated the movement of filamentous ICV on a receptor-fixed glass surface instead of a cell surface. This *in vitro* system was developed for performing imaging analyses of virus movement via the receptor exchange mechanism (1); it allows the high-resolution tracking of a single virus particle and is more suitable than uneven cell surfaces for performing a quantitative analysis of virus motility. We coated the surfaces of glass coverslips with bovine mucin, which is a sialoglycoprotein with Neu5,9Ac2 and acts as a viral receptor (12), and observed the movements of the ICV filamentous strain Ann Arbor/1/50 (AA) and spherical strain Taylor/1233/47 (Taylor) on these mucin-coated surfaces using surface reflection interference contrast microscopy (SRICM) (Fig. 2A).

## RESULTS AND DISCUSSION

SRICM allows the imaging of unlabeled influenza viruses by enhancing the interference of light reflected by an interphase between the glass surface and the virus particle (Fig. 2A and B). Under normal experimental conditions, ICVs moved around the mucin-coated glass surface. However, this ICV movement was completely blocked by treatment with J14, an antibody against the receptor-binding domain in the HEF glycoprotein of the AA virus (Fig. 2C; see Movie S1 in the supplemental material) (13). After addition of this anti-HA antibody, most viruses dissociated from the glass surface. Although a fraction of viruses remained on the surface in the presence of J14, the adhesion areas of the virions to the glass surface decreased and large parts of the virus surfaces dissociated from the glass surface. Most importantly, the virus movement that was observed prior to the antibody addition completely disappeared after its addition. In contrast, treatment with an antibody against the ES domain (K16) caused the virus movement to become slower, but it did not dissociate the viruses from the glass surface (Fig. 2D; see Movie S2 in the supplemental material) (13). The observed inhibitory effect on ICV movement of the anti-ES antibody is similar to the effect of an NA inhibitor on IAV movement, although treatment with an NA inhibitor completely halted virus movement (1). Together, these results demonstrate that ICV moves via an HA-receptor exchange mechanism that is accelerated by ES. Because we found that the pattern of ICV movement was not different at temperatures between 25°C and 37°C, all of the experiments described below were performed at room temperature (25 to 27°C).

**FIG 3:**
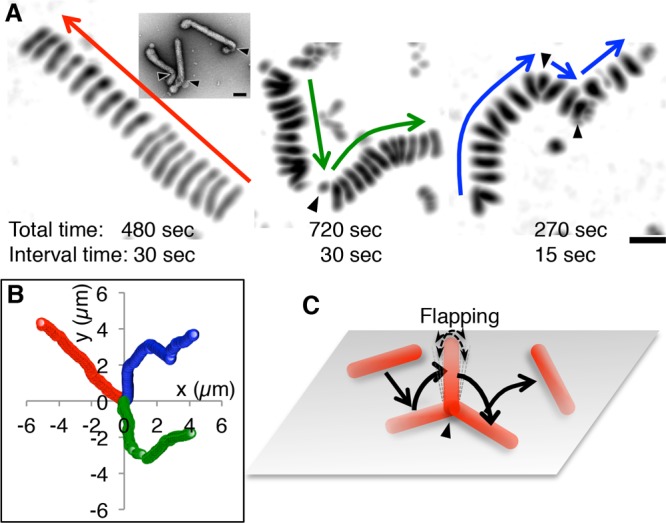
Filamentous ICV movements on a mucin-coated surface. (A) Superimposed images of filamentous ICVs as they move along a mucin-coated surface. Three different viruses are shown. Arrows indicate the directions of viral movements. The viruses either moved straight without turning (left), with occasional turns (middle), or with relatively frequent turns (right). Virus turns are indicated by arrowheads. Bar, 1 μm. (Inset) Electron microscopy (EM) images of negatively stained filamentous AA viruses. Arrowheads indicate bends in the virus filament. Bar, 0.1 μm. (B) Trajectories of the three ICVs shown in panel A. The centers of ICV were plotted every 1 s. The red, green, and blue trajectories correspond to the left, middle, and right ICVs, respectively, in panel A. (C) A schematic illustrating the movement of the filamentous ICV in the middle of panel A. While turning, the filamentous virus stood up, flapped for several seconds, and then flopped down. Thereafter, the virus moved at an angle different from that of its original direction of movement.

Although ICV moves by the same mechanism as IAV, ICV movements are more highly regulated than IAV movements. Filamentous AA viruses moved straight at a constant rate with occasional turns or deformations (Fig. 3A; see Movies S3, S4, and S5 in the supplemental material). Compared with the previously observed random movement of IAV (1), ICV movement was highly oriented in the same direction at least for several minutes (Fig. 3A and B). Before and after deforming or turning, identical virions could be traced continuously. Because turning and deforming events were also observed in experiments using a very low density of viruses, it is unlikely that one virion detached from the surface and another virion subsequently attached to the surface close by during these turning or deforming events.

As seen in Fig. 3A, left panel, the filamentous AA virus appeared to shorten while it moved. Notably, filamentous AA viruses frequently bent at their ends, forming a J shape (Fig. 3A, inset). As such, it is likely that the filamentous virus in Fig. 3A, left panel, did not shorten but rather deformed from its straight form into a J shape. After the deformation, the upper right end of the filament became slightly thick, indicating that this end had bent and consequently increased the adhesion area. After deforming into a J shape, the filamentous virus continued to move straight in the same direction as it had prior to bending, implying that the virus had not moved via simple rolling with a single rotational direction. When filamentous AA viruses turned, the images of virus filaments became smaller (Fig. 3A, middle and right panels), indicating that the adhesion areas of the virus filaments to the surface had decreased. Presumably, while turning, the ICVs stood up, flapped for several seconds, and then flopped down at an angle different from that of the original direction of movement (Fig. 3C; Movies S4 and S5). Interestingly, superimposed images of moving filamentous virus appear to align at distances with the same interval, indicating that the filamentous viruses move at a constant speed (Fig. 3A). Therefore, the filamentous ICVs may regulate not only the direction, but also the speed, of their movement.

**FIG 4:**
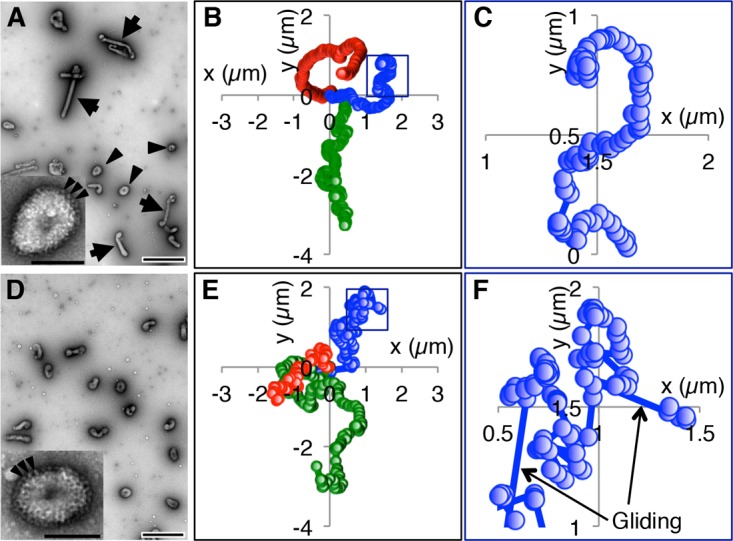
Spherical ICV movements on a mucin-coated surface. (A) Morphology of AA virus. Negatively stained AA viruses were observed by EM. Arrowheads and arrows indicate spherical and filamentous viruses, respectively. Bar, 0.5 μm. (Inset) High-magnification image of a single spherical AA virus. Arrowheads indicate some of the HEF spikes that are densely distributed over the entire virus surface. Bar, 0.1 μm. (B) Trajectories of three spherical AA viruses. (C) Enlarged image of the square in panel B. (D) EM images of Taylor viruses. Bar, 0.5 μm. (Inset) High-magnification image of a single spherical Taylor virus. Arrowheads indicate some of the HEF spikes that are densely distributed over the entire virus surface. Bar, 0.1 μm. (E) Trajectories of three spherical Taylor viruses. (F) Enlarged image of the square in panel E. Arrows indicate rapid (gliding) motions. In panels B, C, E, and F, the centers of the ICVs were plotted every 1 s.

Although the AA virus has a predominantly filamentous morphology, a fraction of the viruses in this strain are spherical (Fig. 4A). To confirm the effect of filamentous morphology on the unidirectional movement of ICV, we examined the movement of spherical AA viruses on mucin-coated surfaces and compared their movement with the movement of the filamentous AA viruses. The spherical AA viruses moved windingly on the mucin-coated surfaces (Fig. 4B and C; see Movie S6 in the supplemental material), unlike the filamentous AA viruses (Fig. 3B), indicating that the unidirectional movement of the filamentous viruses depended on their filamentous morphology. To confirm the contribution of filamentous morphology to unidirectional movement, we next analyzed the movement of the Taylor virus, which, unlike many other ICV strains, has a spherical morphology (Fig. 4D) (14). However, the spherical morphology and HEF distribution of the Taylor virus were similar to those of spherical AA viruses (Fig. 4A and D, insets). The Taylor viruses appeared to move randomly on the mucin-coated surfaces (Fig. 4E and F; see Movie S7 in the supplemental material), confirming that the filamentous morphology of ICV is the cause of its unidirectional virus movement. However, the spherical Taylor virus appeared to move more randomly than the spherical AA virus (Fig. 4B, C, E, and F).

**FIG 5:**
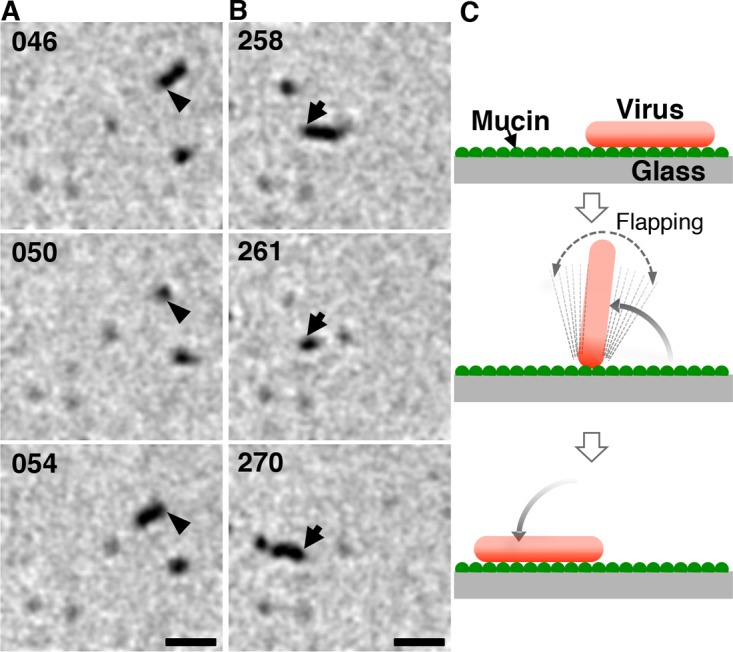
Flapping of a short filamentous Taylor virus. (A, B) Two sequential images of the single filamentous virus in Movie S6 are shown. Arrows and arrowheads indicate the identical ends of the virus filament. For several seconds around 50 s (A) and 261 s (B), the filamentous virus was flapping. The time (in seconds) is indicated in the upper left of each panel. Bar, 1 μm. (C) A schematic illustrating virus flapping.

Interestingly, the random movement of the Taylor virus might not depend on the virus length. A small fraction of Taylor viruses have a short filamentous morphology, and these filamentous Taylor viruses turned frequently by flapping and moved randomly on the mucin-coated surface in a manner similar to that of the spherical Taylor viruses (Fig. 5; see Movie S8 in the supplemental material). Additionally, compared with the spherical Taylor viruses, the spherical AA viruses moved somewhat directionally. Together, these results imply that a separate mechanism distinct from the filamentous morphology contributes to the unidirectional virus movement.

**FIG 6:**
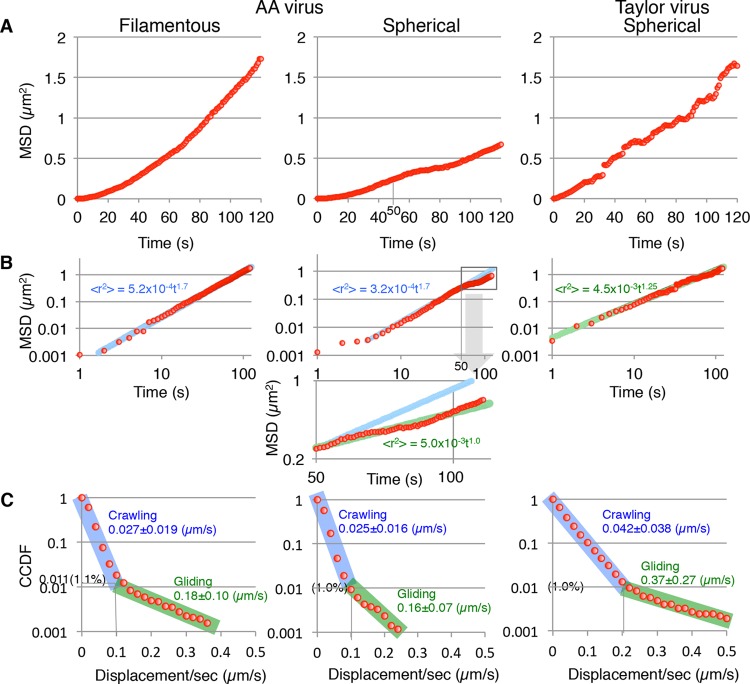
Quantitative analyses of ICV movements. (A) Mean square displacements (MSDs) of the filamentous AA, spherical AA, and spherical Taylor viruses. (B) Log-log plots of MSDs. MSDs were fitted by the equation: ⟨*R*^2^⟩ = *C* × *t^n^*, where ⟨*R*^2^⟩ is the MSD, *C* is the constant, *t* is time, and *n* is a constant between 1 and 2. Fitting curves are presented as blue or green lines. The lower graph is an enlarged graph of spherical AA virus MSD and corresponds to the square in the upper graph. (C) Frequency analyses of crawling and gliding by filamentous AA, spherical AA, and spherical Taylor viruses. The displacements of the viruses per second were analyzed using the complementary cumulative distribution functions (CCDF). Virus crawling and gliding phases are highlighted by blue and green, respectively. Lengths of crawling and gliding are expressed as means ± standard deviations (SD).

A quantitative analysis of ICV virus trajectories demonstrated that the filamentous morphology of ICV does not generate the unidirectional virus movements but rather helps the unidirectional movements to persist. We analyzed the trajectories of filamentous and spherical viruses for long time periods and calculated the mean square displacements (MSDs) from the initial viral attachment positions or from the virus positions at the time recording was begun (Fig. 6A and B). The MSDs of filamentous AA and spherical Taylor viruses resembled a parabolic curve and straight line, respectively, whereas the MSD curve of spherical AA viruses was not a single-phase curve (Fig. 6A). Theoretically, when the viruses move randomly, the MSD obeys the equation for two-dimensional random diffusion: ⟨*R*^2^⟩ = 4*Dt*, where *R* is the displacement of the virus, ⟨*R*^2^⟩ is the MSD, *D* is the diffusion coefficient, and *t* is time (in seconds) (1, 15). In contrast, when the viruses move linearly, the displacement (*R*) of the virus is equal to the product of the velocity (*V*) and time (*t*). Consequently, the MSD instead obeys the equation ⟨*R*^2^⟩ = ⟨*V*^2^⟩*t*^2^, where ⟨*V*^2^⟩ is the mean square velocity. Therefore, the MSD of viruses moving randomly is proportional to the time (*t*), whereas the MSD of viruses moving linearly is proportional to the square of time (*t*^2^). The log-log plots of the MSDs are suitable for a quantitative evaluation of the linearity or randomness of virus movements (Fig. 6B). The MSD of filamentous AA viruses was proportional to *t*^1.7^, indicating that filamentous AA virus movement is more similar to linear movement than to random movement. In contrast, the MSD of spherical Taylor viruses was proportional to *t*^1.25^, indicating that the Taylor virus movement resembles random movement rather than linear movement. Interestingly, the MSD of spherical AA viruses was proportional to *t*^1.7^ within the initial 50 s of movement, but after the first 50 s, the MSD deviated downward from the initial fitting curve (Fig. 6B, middle panel), meaning that the spherical AA viruses move directionally like the filamentous AA viruses for a relatively short period of time (<50 s) and thereafter alter their movement pattern progressively closer to that of random movement. This alteration in movement pattern is not caused by the virus switching from linear motion to random motion at 50 s. Rather, the spherical AA virus makes repeated winding motions from the onset (Fig. 4B and C), which gradually randomize the direction of virus movement. Consequently, the randomness of the virus movement does not become obvious until 50 s. Conversely, the filamentous AA viruses can move directionally for a long period of time. These findings indicate that the filamentous morphology of AA viruses contributes to the persistence of directional movement but does not generate the direction of virus movement. Therefore, the AA viruses have some other mechanism for generating the direction of virus movement.

We hypothesized that the short-range motions specific for AA viruses act to generate the direction of virus movement. Previous work reported that IAV Puerto Rico/8/34 (H1N1) and Aichi/2/68 (H3N2) strains moved randomly with occasional rapid (gliding) motions in addition to their ordinary gradual (crawling) motions (1). Like IAV, the Taylor viruses occasionally moved utilizing rapid motions, whereas the spherical AA viruses moved only in a gradual fashion (Fig. 4C and F). Therefore, the occasional gliding was suspected as the cause of the random movement. To test the effect of gliding on random movement, the displacements of viruses per second were analyzed using the complementary cumulative distribution function (CCDF) (Fig. 6C). The CCDF is defined as follows: $F(r)=\int_{r}^{\infty }p(r)dr$, where *F*(*r*) is the CCDF of *r*, *r* is the displacement of viruses per second, and *P*(*r*) is the frequency function of *r*. The CCDF is more suitable than the “normal” frequency function *P*(*r*) for analyses of rare events, like gliding motions (1). Interestingly, we found that the frequency of gliding for Taylor viruses is 1.0%, which is the same as those for spherical and filamentous AA viruses (Fig. 6C). However, the border length between crawling and gliding of the Taylor virus was 0.2 μm/s, which is equal to those of the IAV subtype H1N1 and H3N2 strains (1) and is twice as large as those of the spherical and filamentous AA viruses (0.1 μm/s). Consequently, the mean gliding length of Taylor viruses (0.37 ± 0.27 μm/s) is more than double those for the spherical AA viruses (0.16 ± 0.07 μm/s) and filamentous AA viruses (0.18 ± 0.10 μm/s). Similarly, the mean crawling length of Taylor viruses might be longer than that of AA viruses, although the crawling phases contained the unmoving state and, therefore, the exact crawling lengths remain unclear. These results indicate that the random movement of the Taylor viruses does not depend on the gliding frequency of these viruses but rather on the lengths of their crawling and gliding movements, which are longer than those of the AA viruses. Conversely, the relatively short-range crawling and gliding motions employed by AA viruses likely generate the direction of virus movement.

**FIG 7:**
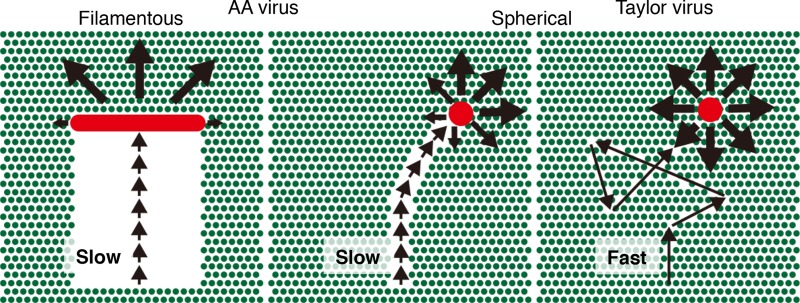
Dual regulation mechanisms of ICV directional movement. A spherical Taylor virus (red circle) moves quickly on the surface utilizing long crawling and gliding (right). During fast movement, viral ESs degrade only a small amount of receptor molecules (green circles) along the virus trajectory. After virus movement, most receptors remain around the virus and allow the virus to move in various directions, resulting in the random movement of the Taylor virus. In contrast, a spherical AA virus (red circle) moves slowly with short crawling and gliding, and viral ESs degrade a relatively large number of receptors along the trajectory (middle). Consequently, the virus tends to move forward. In a filamentous AA virus (red rod), the directional movement persists for a long period of time due to the filamentous morphology of the virus (left). Because the receptor molecules behind the virus filament are broadly degraded, the distinct difference in receptor density between the front and the back of the virus filament fixes the direction of virus movement.

Short crawling and gliding in ICV presumably generate directional movement through the degradation of receptors by viral ESs (Fig. 7). Because short crawling and gliding make a virus particle move slowly, the viral ESs efficiently degrade receptor molecules along the pathway traveled by the virus, consequently making the virus move forward. Thus, short crawling and gliding result in directional movement. In contrast, because long crawling and gliding make a virus move quickly and the virus spends very little time in each location, the ESs only minimally degrade the receptors on the virus trajectory. This permits most receptors to remain around the virus particle, allowing the virus to move in various directions, including backward, and results in virus movement that resembles random movement. Although ES catalytic activity might be involved in allowing directional movement, higher ES activity may not cause higher directional movement. A comparative analysis of previously reported IAV subtypes and NA mutant IAVs suggests that higher NA activities do not affect the random movement of IAV but rather increase the gliding frequency (1). Furthermore, the cooperation of HA and NA was proposed to generate the movement pattern of IAV. Similarly, a functional balance of ES activity and HA affinity in the AA virus regulates the lengths of crawling and gliding to be short, and this consequently generates directional movement. To elucidate the functional balances of ES and HA, further experiments that comparatively analyze the motilities of mutant ICVs with manipulated ES activities or HA affinities are required.

In the filamentous AA virus, directional movement persists over a long period of time due to the filamentous morphology of the virus (Fig. 6B and 7). Notably, the filamentous morphology of ICV depends on the virus matrix protein, CM1 (14, 16, 17). Therefore, the ES and HA of HEF generate the direction of the virus movement, whereas CM1 enables the unidirectional movement of filamentous ICVs to persist, although its contribution is indirect.

The energy source of ICV unidirectional movement is likely the chemical energy of the receptor molecule. IAVs move randomly on receptor-fixed surfaces, and the IAV random movement utilizes the impacts of water molecules as an energy source in the same manner as Brownian motion and does not need any other energy (1). However, the filamentous ICV movement is highly regulated in both direction and speed and therefore needs an energy source other than the impacts of water molecules, such as ATP. We propose that ICV utilizes the chemical energy of the ester bond in receptor molecules as the driving energy for its unidirectional movement. Viral ES cleaves the acetyl group at the C-9 position from the terminal Neu5,9Ac2 of the virus receptor oligosaccharide (11). The chemical bond energy released from the Neu5,9Ac2 hydrolysis is converted into the receptor density difference between the front and the back of the virus filament on the surface. Thereafter, the receptor density difference is utilized as the mechanical energy for the unidirectional movement of filamentous ICV. This mechanochemical coupling of the chemical bond energy to the unidirectional movement of ICV utilizing the density difference of receptor molecules as an energy-intermediate is a novel energy transfer mechanism different from those in the known motile machineries of eukaryotic and bacterial cells. To demonstrate this novel energy transfer mechanism, direct evidence for the receptor density difference around a virion is needed. Achatinin H, an uncommon lectin from the snail *Achatina fulica*, specifically binds to Neu5,9Ac2-terminated oligosaccharide (18). Further experiments employing such specific probes are required to demonstrate the hypothesized receptor density difference.

Although influenza virus motility based on the receptor exchange mechanism is important for virus infection (1), the biological significance of unidirectional ICV movement remains unclear. ICVs preferentially infect the epithelial cells of human upper respiratory tracts (19). Moreover, freshly isolated influenza viruses from human upper respiratory tracts were reported to predominantly have filamentous morphology (20, 21). These findings imply that the filamentous viruses have advantages for infecting the epithelial cells of human upper respiratory tracts. Filamentous IAV enters into a host cell through macropinocytosis, and IAV binding to a cell surface membrane induces macropinocytosis in cultured cells (6). However, it is unlikely that virus entry occurs over entire cell surfaces *in vivo*. Virus entry in the respiratory tract may occur on specific cells or cell surface domains. Cilium surfaces that occupy the predominant areas of respiratory tract surfaces might lack endocytosis activity because the insides of cilia are occupied by microtubule bundles and have no space for endocytosis invagination (22). The unidirectional movements of ICV likely have advantages for quickly moving away from those surfaces that are not active in endocytosis.

Unfortunately, the receptor distribution and density *in vivo* are currently unknown, so it was not possible to intentionally mimic these conditions in our study. The potential differences in receptor distribution and density *in vivo* may affect virus motility in a living host.

In conclusion, like IAV, ICV also has motility that is driven by the receptor exchange mechanism. However, in contrast to IAV random movement, filamentous ICV movement is highly regulated in direction both by its characteristic motility, which employs short-range crawling and gliding motions, and by its filamentous morphology; these characteristics, respectively, allow the generation and the persistence of the unidirectional movement of the virus. Both types of regulation are based on the receptor density gradient surrounding the virus. While the virus is moving, ES degrades the receptors along the trajectory of the virus and consequently produces the receptor density gradient around the virus. Through HA sensing the receptor density gradient generated by ES, the filamentous ICV avoids moving backward, acting as if it has a memory of where it has traveled. We propose that, in addition to its own motile machinery, influenza virus potentially also has a unique direction control mechanism for virus motility.

## MATERIALS AND METHODS

### Viruses.

The ICV strains Ann Arbor/1/50 and Taylor/1233/47 were each grown in the amniotic cavity of 8-day-old embryonated hen eggs and purified by differential centrifugation and sucrose density gradient centrifugation (23).

### Imaging virus movement.

For the imaging analyses of virus movements, the glass bottoms of culture dishes (Matsunami, Kishiwada, Japan) were coated with bovine mucin (1 mg/ml; Sigma-Aldrich, St. Louis, MO, USA), which was dissolved in phosphate-buffered saline (PBS) and filtered through a polyvinylidene fluoride membrane filter with a 0.22-μm pore (Millipore, Billerica, MA, USA) to remove insoluble mucin, and the dishes were then incubated for 1 h at room temperature. The glass surface of each dish was washed twice with PBS to remove any unbound mucin. This cycle of coating and washing was repeated twice. To examine virus movement, viruses diluted in PBS were added to the mucin-coated glass surfaces and observed by SRICM (Nikon, Tokyo, Japan) using a 100× lens objective (APO TIRF; numerical aperture, 1.49). Virus images were acquired every 1 s for a total duration of 15 min. Images of 50 filamentous AA, 30 spherical AA, and 30 spherical Taylor viruses were analyzed. Virions with lengths of more than 0.5 μm were regarded as filamentous, whereas virions with diameters of less than 0.3 μm were regarded as spherical.

### Analysis of virus movement.

Images were analyzed with ImageJ software (http://rsb.info.nih.gov/ij/). The positions (*x* and *y* coordinates) of viruses were determined as the centers of darkness. To test the stability and accuracy of our apparatus, IAV particles were fixed on a coverslip coated with fetuin (1 mg/ml) in the presence of an NA inhibitor (zanamivir; GlaxoSmithKline Research and Development Ltd., Stevenage, UK), and time-lapse images of IAV particles were acquired (1). The displacement of fixed particles was observed to vary by less than 20 nm per 1 s (corresponding to one image frame), which is comparable with published values from earlier studies (1, 24, 25).

## Supplementary Material

Supplemental material
