# Supplementary material for: Unique Directional Motility of Influenza C Virus Controlled by Its Filamentous Morphology and Short-Range Motions
Source: J Virol. 2018 Jan 2;92(2):e01522-17. doi: 10.1128/JVI.01522-17 (PMC5752937; doi:10.1128/JVI.01522-17)
Supplement: Supplemental material [file supp_92_2_e01522-17__index.html]

Unique Directional Motility of Influenza C Virus Controlled by Its Filamentous Morphology and Short-Range Motions — Supplemental material 

# Unique Directional Motility of Influenza C Virus Controlled by Its Filamentous Morphology and Short-Range Motions

## Supplemental material

- Supplemental file 1 -

  Supplemental Movie Legends.

  PDF, 61K
- Supplemental file 2 -

  Movie S1 (Effect of anti-HA antibody on ICV movements.)

  MOV, 2.4M
- Supplemental file 3 -

  Movie S2 (Effect of anti-ES antibody on ICV movements.)

  MOV, 7.0M
- Supplemental file 4 -

  Movie S3 (A filamentous AA virus moves straight on a mucin-coated glass surface without turns.)

  MOV, 1.9M
- Supplemental file 5 -

  Movie S4 (A filamentous AA virus moves on a mucin-coated glass surface with occasional turns.)

  MOV, 1.3M
- Supplemental file 6 -

  Movie S5 (A filamentous AA virus moves on a mucin-coated glass surface with frequent turns.)

  MOV, 947K
- Supplemental file 7 -

  Movie S6 (Spherical AA viruses move windingly on a mucin-coated glass surface.)

  MOV, 1.5M
- Supplemental file 8 -

  Movie S7 (Spherical Taylor viruses move randomly on a mucin-coated glass surface.)

  MOV, 1.4M
- Supplemental file 9 -

  Movie S8 (A short filamentous Taylor virus moves randomly on a mucin-coated glass surface.)

  MOV, 722K
