# Supplementary material for: Unique Directional Motility of Influenza C Virus Controlled by Its Filamentous Morphology and Short-Range Motions
Source: J Virol. 2018 Jan 2;92(2):e01522-17. doi: 10.1128/JVI.01522-17 (PMC5752937; doi:10.1128/JVI.01522-17)
Supplement: Supplemental material [file JVI.01522-17_zjv002183251s9.pdf]

Unique directional motility of influenza C virus controlled by its filamentous morphology and short-range motions

Supplemental material

- **Supplemental file 1** -
  Movie S1. (Effect of anti-HA antibody on ICV movements. Timing of the antibody addition is indicated by the white circle in the upper left. This movie plays at 50× speed. Scale bar = 1 μm.)
  MOV(H.264), 2.5M
- **Supplemental file 2** -
  Movie S2. (Effect of anti-ES antibody on ICV movements. Timing of the antibody addition is indicated by the white circle in the upper left. This movie plays at 50× speed. Scale bar = 1 μm.)
  MOV(H.264), 7.3M
- **Supplemental file 3** -
  Movie S3. (A filamentous AA virus moves straight on a mucin-coated glass surface without turns. This movie plays at 100× speed. Scale bar = 1 μm.)
  MOV(H.264), 2.0M
- **Supplemental file 4** -
  Movie S4. (A filamentous AA virus moves on a mucin-coated glass surface with occasional turns. This movie plays at 100× speed. Scale bar = 1 μm.)
  MOV(H.264), 1.4M
- **Supplemental file 5** -
  Movie S5. (A filamentous AA virus moves on a mucin-coated glass surface with frequent turns. This movie plays at 100× speed. Scale bar = 1 μm.)
  MOV(H.264), 1.0M
- **Supplemental file 6** -
  Movie S6. (Spherical AA viruses move windingly on a mucin-coated glass surface. This movie plays at 100× speed. Scale bar = 1 μm.)
  MOV(H.264), 1.6M
- **Supplemental file 7** -
  Movie S7. (Spherical Taylor viruses move randomly on a mucin-coated glass surface. This movie plays at 100× speed. Scale bar = 1 μm.)
  MOV(H.264), 1.5M

- **Supplemental file 8** -
  Movie S8. (A short filamentous Taylor virus moves randomly on a mucin-coated glass surface. This movie plays at 100× speed. Scale bar = 1 μm.)
  MOV(H.264), 0.7M
